# Individual‐Level Trait Responses in Cyanobacterial Populations and Communities

**DOI:** 10.1111/ele.70348

**Published:** 2026-02-23

**Authors:** Arnaud P. Louchart, Annemieke M. Drost, Chaohong Lin, Suzanne M. H. Naus‐Wiezer, Zhipeng Duan, Elena Litchman, Dedmer B. Van de Waal

**Affiliations:** ^1^ Department of Aquatic Ecology Netherlands Institute of Ecology (NIOO‐KNAW) Wageningen the Netherlands; ^2^ Department of Freshwater and Marine Ecology, Institute for Biodiversity and Ecosystem Dynamics University of Amsterdam Amsterdam the Netherlands; ^3^ Institute of Water Science and Technology Hohai University Nanjing Jiangsu China; ^4^ Kellogg Biological Station Michigan State University Hickory Corners Michigan USA

**Keywords:** cyanobacteria, functional assessment, functional diversity, functional traits, *Microcystis* sp., multi‐traits, natural communities, trait‐based ecology

## Abstract

Trait‐based approaches support the mechanistic understanding of individual organism responses to resource availabilities that underlie population and community dynamics. For microbes such as phytoplankton, however, it remains challenging to obtain individual cell traits, particularly in natural communities. Here, we provide a flow cytometry‐based approach using a freshwater cyanobacterium *Microcystis* spp. culture and assessed individual‐level trait responses to nitrogen, phosphorus and light limitation and high *p*CO_2_. Then, these responses served as ‘fingerprints’ to describe the main drivers in natural cyanobacterial communities. We observed distinct responses in multidimensional trait space, that is, the integrated phenotype, which differed particularly between nitrogen and light limitation. Notably, cellular contents of the pigments phycocyanin and chlorophyll‐a decreased with nitrogen limitation and increased with light limitation, which was confirmed in natural communities. Overall, our results show how individual‐trait responses to known environmental conditions can be used to understand natural cyanobacterial population and community dynamics.

## Graphical abstract

Trait‐based approaches can elucidate mechanisms underlying dynamics of phytoplankton population and community, but assessing individual‐level traits in natural systems remains challenging. This study used flow cytometry to quantify single‐cell trait responses of Microcystis spp. under nutrient, light and CO_2_ experiment, and these responses were applied as fingerprints to identify key drivers in natural communities. Distinct integrated phenotypes, particularly under nitrogen and light limitation, revealed contrasting trait responses relevant to the dynamics of cyanobacterial populations and communities.

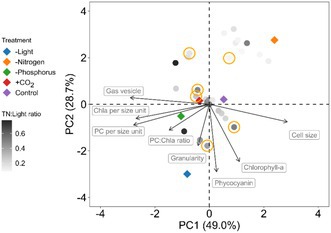

## 1 Introduction

Trait‐based ecology offers a valuable framework for understanding the mechanisms shaping biodiversity and ecosystem functioning. Trait‐based approaches involve functional traits that represent morphological, physiological, life‐history and behavioural characteristics, which play a crucial role in determining individual performance under specific abiotic and biotic conditions (Litchman and Klausmeier 2008; McGill et al. 2006). Consequently, variability in functional traits influences species interactions, food‐web dynamics and biogeochemical cycles that, in turn, underlie ecosystems functioning (Chacón‐Labella et al. 2023; Fontana et al. 2021; Litchman et al. 2021).

Historically, trait‐based ecological studies focused on variation across species rather than within species (McGill et al. 2006). Yet, we know that traits can vary substantially within single species populations, which may affect ecological dynamics (Fontana et al. 2014; Violle et al. 2012). Trait variation within populations arises from differences between genotypes (Brandenburg et al. 2018), which determine a population's adaptive evolutionary responses. Moreover, variation within a genotype (phenotypic plasticity) determines how an individual genotype can adjust to changing environments (Forsman 2015; Pigliucci et al. 2006).

Organisms have many traits and respond to changing environmental conditions. The multi‐trait response to environmental changes refers to the multidimensional trait space of one or more genotypes (Rosenfeld 2002), while the variation across individuals within this trait space illustrates the intraspecific variation in traits. Defining a phenotype from such a multidimensional trait space is complex yet may be simplified through dimension reduction methods that preserve trait values and interactions. Such a reduced‐dimensional representation of trait space is called the integrated phenotype (Levine et al. 2024), which can be described with functional diversity indices (Mason et al. 2005, Figure 1). For instance, functional richness measures the trait space size, reflecting the variety of trait combinations. Functional evenness indicates how evenly distributed individuals are within the trait space, while functional divergence measures how much traits deviate from the centroid of the functional space, revealing how individuals differ from the population mean.

**FIGURE 1:**
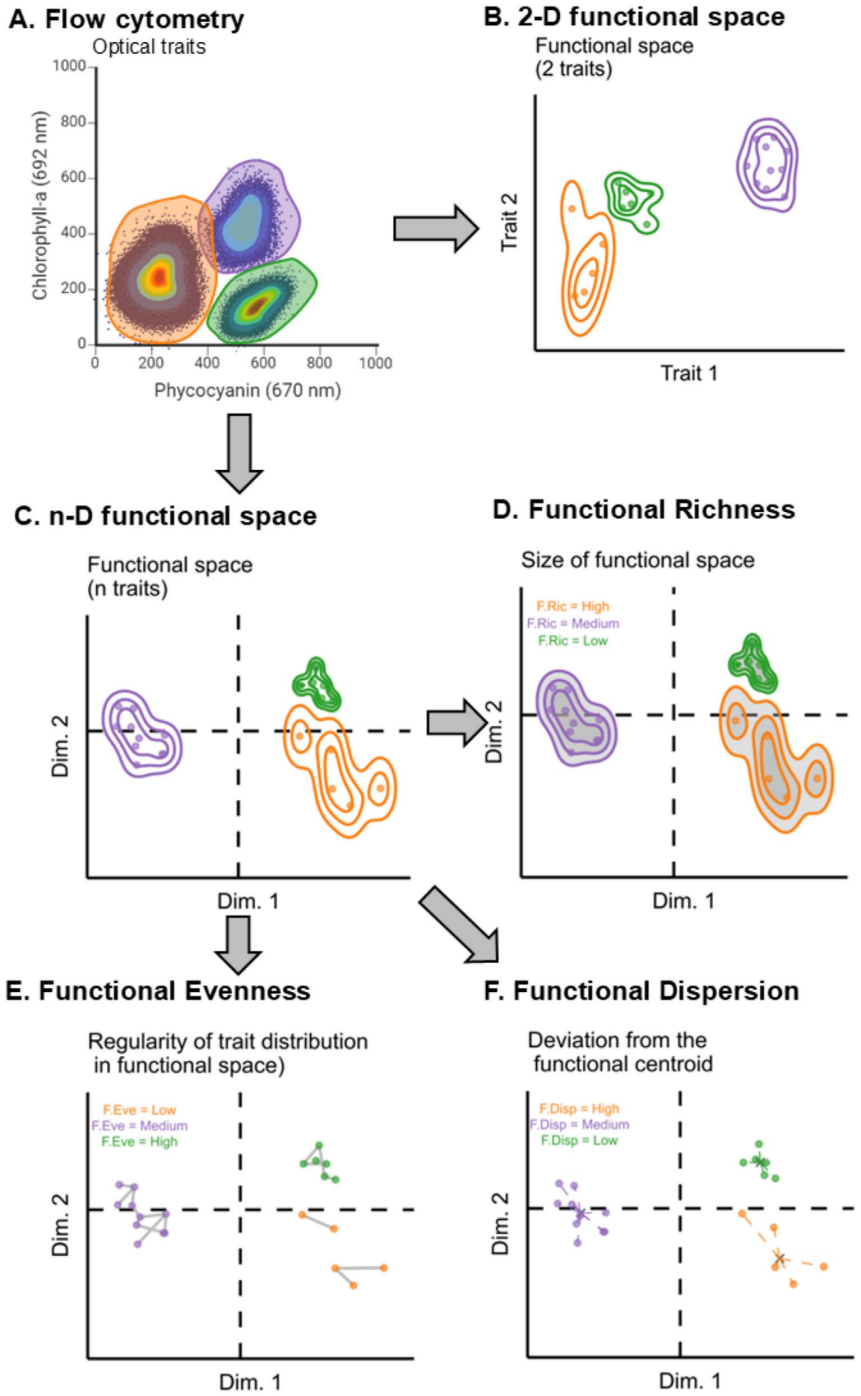
Stepwise conversion of flow cytometry optical traits into functional diversity indices to assess the responses of species under different pressures in ecological studies. (A) Flow cytometry data of two optical traits (670 nm and 692 nm) and their associated functional traits (phycocyanin and chlorophyll‐a, respectively). (B) Distribution of the traits in the 2‐dimensions standardised functional space. (C) Distribution of the traits in the n‐dimensions standardised functional space (PCA reduction + hypervolume computation). Functional diversity indices computed on the n‐dimensions functional space, where: (D) Functional richness (F. Ric) measures the size of the functional space defined by the traits. (E) Functional evenness (F. Eve) reflects the regularity of trait distribution within the functional space. (F) Functional dispersion (F. Disp) reflects the deviation of the traits from the centroid of the functional space. Different colors were used to illustrate three different treatments and their functional responses. Panel (A) was created with BioRender.

Assessing multiple traits at the individual level increases mechanistic understanding of population dynamics, as trait responses at the individual level underlie changes in populations and communities (Bolnick et al. 2011; Des Roches et al. 2018; Violle et al. 2012). In phytoplankton ecology, traditional measurements rely on mean trait values in populations, which overlook intraspecific variation and limit our understanding of population dynamics (Wong and Carmona 2021). Estimating traits at the individual level is labor‐intensive, particularly in phytoplankton ecology, where it often involves time‐consuming methods like microscopy (Argyle, Hinners, et al. 2021). Flow cytometry enables rapid assessment of various individual‐level traits through the measurement of light scattering, absorption and emission (Pomati et al. 2011). This technique is well suited for phytoplankton because cells exhibit pigment autofluorescence, which, together with light scatter, characterises several traits simultaneously (Table 1). Light scattering estimates cell size, shape and complexity, while absorption and emission indicate pigment composition and contents (Trask et al. 1982). Flow cytometry allows assessment of individual‐level functional responses to environmental gradients and, thereby, of the integrated phenotype, thus scaling from individual cells to population dynamics and community assembly (Chacón‐Labella et al. 2023; Fontana et al. 2021). However, the drivers of the integrated phenotype can only be understood through phytoplankton culture experiments under different conditions.

**TABLE 1:** List of flow cytometry optical traits related to functional traits, their ecological relevance and underlying mechanisms. FSC indicates Forward Scatter, and SSC Sideward Scatter.

FCM optical trait	Category^a^	Functional trait (labeling used)	Ecological relevance	Underlying mechanism
FSC^b^	Morphological	Cell Size (cell size)	Nutrient uptake; sinking rate	Surface‐to‐volume ratio governs nutrient uptake; Large cells sink faster.
SSC^b^	Morphological	Granularity (Granularity)	Internal organisation related to storage	Storage of C, N and P related compounds
Fill SSC^c^	Physiological	Gas vesicle (Gas vesicle)	Buoyancy regulation	Vertical positioning in the water column, optimization of light and nutrient access
670/40 (640 nm)^d^	Physiological	Phycocyanin (Phycocyanin)	Spectral niche exploitation	Accessory pigment synthesis regulated by light and nitrogen availability
670/40 (640 nm):FSC^e^	Morphological and physiological	Phycocyanin per size unit (PC per size unit)	Phycocyanin efficiency	Ability to capture light in red and orange wavelengths
692/30 (488 nm)^b^	Physiological	Chlorophyll‐a (Chlorophyll‐a)	Photophysiological status	Pigment synthesis regulated by light and nitrogen availability
692/30 (488 nm):FSC^f^	Morphological and physiological	Chlorophyll‐a per size unit (Chla per size unit)	Chlorophyll‐a efficiency	Ability to capture light in red and blue wavelengths
670/40 (640 nm):692/30 (488 nm)^d^	Physiological	Phycocyanin versus chlorophyll‐a ratio (PC_Chla)	Photophysiological strategy	Relative pigment investment; pigment balance regulation

^a^ Litchman and Klausmeier (2008).

^b^ Gill et al. (2022).

^c^ Walsby (1994).

^d^ Collier (2000).

^e^ This study.

^f^ Argyle, Hinners, et al. (2021); Argyle, Walworth, et al. (2021).

Here, we propose an approach to first measure individual cell responses to environmental variations in a culture experiment and, then use these data to identify key environmental drivers of natural phytoplankton communities. To this end, we used *Microcystis* spp., a colonial, cosmopolitan cyanobacterium capable of forming dense blooms with unicellular, spherical‐shaped cells with a diameter of 1–9 μm (Komárek and Komárková 2002). While blooms of *Microcystis* occur in eutrophic systems, their high biomass often causes light and nutrient limitation. Therefore, we first characterised the integrated phenotype from individual‐level trait responses of a single *Microcystis* strain exposed to resource limitations (i.e., nitrogen, phosphorus and light), and to high *p*CO_2_ and compared them to traditional approaches measuring mean trait responses. Previous work showed that limiting environmental factors affected traits following their elemental demands and physiological requirements, while responses to elevated *p*CO_2_ did not differ from control conditions. For instance, chlorophyll‐a and phycocyanin, which require nitrogen, decreased under nitrogen and phosphorus limitation, but increased under light limitation to optimise light capture (Duan et al. 2021). Gas vesicles, composed of proteins, decreased under nitrogen limitation and increased under phosphorus and light limitation. Cell size consistently increased under all three limiting parameters reflecting arrested growth and accumulation of organic carbon (Duan et al. 2021). After establishing the integrated phenotype using the single *Microcystis* strain, we subsequently projected the obtained individual‐level trait responses onto a natural cyanobacterial community dominated by *Microcystis* to assess key environmental drivers of bloom dynamics. We hypothesize that the individual‐level trait response follows the overall mean responses, with nitrogen and phosphorus limitation reducing pigment content, cell size and gas vesicle content, light limitation increasing pigment and gas vesicle content and high *p*CO_2_ not showing distinct responses. Therefore, we expect these stressors to produce distinct integrated phenotypes, showing similar phenotypes under nitrogen and phosphorus limitation, and a contrasting phenotype under light limitation. Lastly, we expect natural cyanobacterial communities dominated by *Microcystis* to exhibit comparable integrated phenotypes to those observed in *Microcystis* culture, depending on nutrient and light conditions.

## 2 Material and Methods

### 2.1 Experiments

#### 2.1.1 Experimental Design

The data were obtained during experiments reported in Duan et al. (2021). In short, a colony‐forming *Microcystis* strain was isolated from Meiliang Bay, Lake Taihu, in August 2016. Isolation and purification followed the procedure described by Duan et al. (2018, Data S1). The experiments were conducted in 500‐mL Erlenmeyer flasks in modified WC medium at control conditions (33 μmol photons m^−2^ s^−1^ light, 6000 μmol L^−1^ NO_3_^−^, 300 μmol L^−1^ PO_4_^3−^ and 350 μatm *p*CO_2_), and treatments included low light (5 μmol photons m^−2^ s^−1^), low nitrogen (100 μmol L^−1^ NO_3_^−^), low phosphorus (3.5 μmol L^−1^ PO_4_^3−^) and high *p*CO_2_ (1000 μatm) with four replicates (*n* = 4). After acclimation (6–10 days; four generations), cultures reached early stationary phase, and samples were taken for mean population traits (cell size, gas vesicle content, phycocyanin and chlorophyll‐a content). Colonies were broken down into single cells using 100‐fold times dilution with deionised water (Duan et al. 2018). Nitrogen, phosphorus and light limitations were confirmed based on the reduced growth rates and biomass build‐up as compared to control conditions. During the experiment, samples were taken to assess individual‐level traits by flow cytometry. 2 mL aliquots were fixed with a paraformaldehyde‐glutaraldehyde solution (6.75/1) used at a final concentration of 1% (v/v) for flow cytometry analyses. Samples were filtered through a membrane of 70 μm prior to flow cytometry analyses due to the tip size.

#### 2.1.2 Flow Cytometry

We used a BD Influx flow cytometer (BD Biosciences, San Jose, California, U.S.A.) equipped with five lasers (355, 457, 488, 532 and 640 nm) to capture optical information at the individual level. In this study, individual level refers to individual particle‐level measurements, where each event detected by the flow cytometer represents a single cell or a small colony. Fluorescence is measured with laser‐specific filters, and forward and sideward scatter to measure light deviation at small and large angles, respectively. All measurements are based on peak height and reflect internal and external morphophysiological properties at the individual level, some of which are ecologically relevant, and we consider these as functional traits. The individual‐level data were retrieved using high‐throughput automated pipeline (Louchart and de Van Waal 2025), enabling reliable capture of intraspecific phytoplankton trait variation.

#### 2.1.3 Clustering Method

Recognition of *Microcystis* cells was achieved by unsupervised clustering, via the pipeline (Louchart and de Van Waal 2025). We used the HDBSCAN algorithm, which estimates the densities across multiple dimensions (i.e., one dimension per parameter) and identifies high densities regions (Figure S1). The algorithm is performed on log_10_(x + 1) transformed data to reduce the skewness towards the right (Figure S2). The original values were retrieved by back transformation after the clustering procedure.

#### 2.1.4 Individual‐Level Optical Traits

We obtained 18 optical traits at the individual‐level using flow cytometry and derived five additional traits by combining existing ones. We analysed collinearity between optical traits (*r* > 0.8; Figure S3) to reduce the overall redundancy. An additional check was systematically performed to keep all ecologically relevant optical traits. For example, despite the strong correlation between chlorophyll‐a and phycocyanin fluorescence, both traits have different ecological meanings, and we therefore kept them both. This selection reduced the optical traits from 23 to eight functional traits (Table 1). These include Forward Scatter (FSC), indicating cell or colony size (1), Sideward Scatter (SSC) or granularity (2), used as a proxy of internal composition and as proxy for gas vesicles of the cell in cyanobacteria. The SSC was used to define the ‘Fill SSC’ (3) by dividing the cell volume by the granularity, to represent the proportion of the cell volume occupied by gas vesicles as it is usually the largest internal structure in *Microcystis* (Brookes 2000). The fluorescence obtained at 670/30 nm after excitation at 640 nm represents the phycocyanin fluorescence per cell (4). The fluorescence obtained at 692/40 nm after excitation at 488 nm represents the chlorophyll‐a fluorescence per cell (5). Both phycocyanin and chlorophyll‐a fluorescence can be normalised to size (i.e., FSC), which then represent phycocyanin content (6) and chlorophyll‐a content (7) as the standardised proportion of each pigment available for photosynthesis. Lastly, we derived the ratio between phycocyanin to chlorophyll‐a fluorescence (8).

Prior to analysis, each functional trait was log_10_‐transformed and z‐standardised. ANOVA was used to test differences in individual traits across treatments. Functional traits responses were clustered into five treatment groups, while the number of functional clusters was determined by k‐means clustering using the elbow method (Figure S4). We performed a principal component analysis for defining the integrated phenotype (Levine et al. 2024). This method is suitable for continuous and standardised trait data, effectively reducing dimensionality while retaining most of the variance for identifying the integrated phenotypes.

### 2.2 Field Data

#### 2.2.1 Lake Grote Plas

Natural communities were sampled from Lake Grote Plas in the Netherlands (Lat: 52.0210803; Long: 4.3776454). Lake Grote Plas is a shallow (2.8 m average depth) eutrophic lake with recurrent cyanobacterial blooms in summer dominated by *Microcystis* spp., *Dolichospermum* spp. and *Aphanizomenon* spp. (de Waal et al. 2018). The lake was sampled weekly from 12 April 2023 to 25 October 2023 (28 samples). Samples were taken for flow cytometry (as in Section 2.1.1.), as well as for total nitrogen (TN) and total phosphorus (TP). Moreover, in situ measurements were performed for light intensity. Detailed sampling and analyses are described in Appendix S1. Clustering and data extraction at the individual level were processed using Floreada software. Bioassay experiments were conducted to assess nitrogen and phosphorus limitation in lake samples under controlled light and temperature conditions. Treatments included control, nitrogen addition, phosphorus addition and combined nitrogen plus phosphorus addition, with nutrient concentrations selected to stimulate phytoplankton growth. Chlorophyll‐a fluorescence, as a biomass proxy, was obtained by the PhytoPAM (Walz GmbH) on days 0 and 4. Chlorophyll‐a fluorescence characterises the physiological state of the communities and captures the growth limitation (Appendix S2).

### 2.3 Numerical Analyses

Statistical analyses and graphical outputs were performed using R 4.3.1.

#### 2.3.1 Functional Diversity of *Microcystis*

The functional space reveals the relationships between traits using the n‐dimensional hypervolume approach, which considers gaps in the trait distribution and reduces outlier importance compared to traditional methods (Blonder et al. 2014). Thus, the functional space represents the integrated phenotype. PCA eigenvalues were used to build the functional space of each treatment. The two first dimensions which accounted for most variance were used to build the functional space based on their significance using the ‘PCAtest’ function in the R package PCAtest (Camargo 2022). The functional space (i.e., across treatments) and subspace (i.e., within each treatment) of *Microcystis* were computed using the Gaussian kernel density estimation method, which assumes a constant probability density throughout the distribution (Mammola and Cardoso 2020). The kernel bandwidth was automatedly estimated for each PCA dimension using the ‘estimate_bandwidth’ function. We set the quantile threshold to 95% to delineate the functional spaces. All analyses were performed with the hypervolume R package.

To assess trade‐offs, we looked at the pattern of co‐variation within the PCA (Levine et al. 2024). This was possible as PCA preserves the relationship between the variables while it synthesises individual responses across multiple traits by reducing dimensionality and maximising the variance (Argyle, Walworth, et al. 2021). Thereby, irrespective of how data are integrated, the negative associations will be maintained and indicate a possible trade‐off.

Functional diversity incorporates complementary metrics (functional richness, functional evenness and functional dispersion) to characterise the properties of a functional space and the distribution of individuals in it (Figure 1). We used the BAT package to obtain these metrics (Cardoso et al. 2015). We estimated the size of the functional space (i.e., functional richness) using the ‘kernel. alpha’ function. High functional richness indicates that the community occupies a wide range of functional trait space, whereas low richness reflects an occupation of a narrow portion of trait space. The functional evenness, that is, the regularity in the distribution of individuals within the trait space (Mason et al. 2005), is calculated using the function ‘kernel. evenness’. High evenness indicates a regular distribution of individuals within the functional space, while low evenness indicates a clustered distribution in some regions and gaps in others. The functional dispersion, that is, the average distance of the individuals from the centroid of the functional space, represents the capacity of the species to respond to environmental disturbance. High dispersion indicates a better ability for this while low dispersion indicates a poor ability. Functional dispersion was calculated using the function ‘kernel. dispersion’ (Mammola and Cardoso 2020).

We also assessed the variability of *Microcystis* functional diversity within and across the treatments. Due to small sample size (*n* = 20) and a non‐normal distribution, we applied a Kruskal–Wallis test to compare the functional diversity between treatments. Conover‐Iman test with a Benjamini‐Hochberg correction was applied for post hoc pairwise comparisons. The significance threshold of the Conover‐Iman test was set at 0.01.

Lastly, we computed Jaccard similarity to examine the origin of variability between treatments. Jaccard's similarity index provides the proportion of the *Microcystis* functional space shared between two treatments. High similarity indicates a large overlap between the functional spaces, while a low similarity indicates a low overlap between the functional spaces. Thus, low similarity suggests multi‐trait diversification (i.e., production of new functional space). The hypervolume R package contains an extension of the Jaccard index for n‐dimension hypervolumes. The index is produced using the function ‘hypervolume_set’ and is accessed through the ‘hypervolume_overlap_statistic’ function (Blonder et al. 2014).

#### 2.3.2 Functional Assessment of Field Data

For comparison, the functional traits obtained at the individual level of the natural communities were aligned with those obtained from the culture experiments. For this, we established a subset of the natural communities by targeting only cyanobacterial particles of sizes between 0.5 and 10 μm (i.e., to prevent large colonies or aggregates) according to a set of beads of known size. Very few small colonies may still fall within this range, but their influence is minimised, and the subset largely reflects single cells. As flow cytometry is ataxonomic and discriminates particles based on their properties, *Microcystis* could not be discriminated from other cyanobacteria sharing similar optical signatures within the same size range. The subset data were then transformed and normalised following the same procedure as described in Section 2.1.4. A PCA was performed using the functional responses at the individual level of the culture and the field data together. This procedure defines a functional assessment of the natural communities combined with the culture of *Microcystis* to determine the key environmental drivers of the cyanobacterial functional responses in lake Grote Plas.

## 3 Results

### 3.1 Flow Cytometry Functional Traits

**FIGURE 2:**
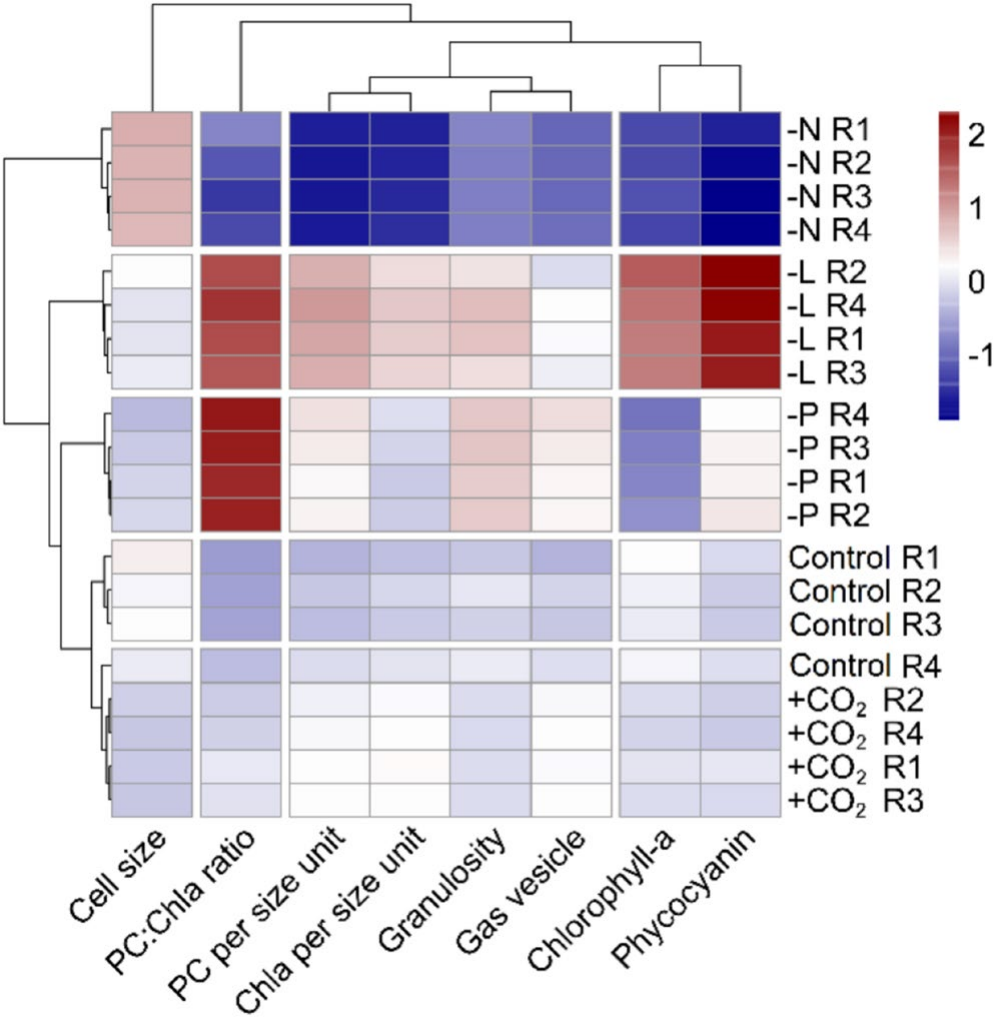
Heatmap of *Microcystis* mean functional trait responses to treatment conditions. The treatments include Control (Cont), high *p*CO_2_ (+CO_2_) and limitation by nitrogen (−N), phosphorus (−P) and light (−L). Treatments are indicated by numbers (R1‐4). Functional traits are displayed in columns, where PC refers to phycocyanin and Chla to chlorophyll‐a in the cells (see also Table 1).

The response of the eight functional traits across the treatments resulted in five treatment clusters and four functional clusters (Figure 2). Each treatment produced a unique response, except for one replicate from the control that was grouped in the high *p*CO_2_ treatment. This reflected the similarity in functional response between the high *p*CO_2_ treatment and control conditions.

The first functional cluster was defined by the granularity (SSC), the gas vesicle content (Fill SSC), phycocyanin and chlorophyll‐a content. These traits strongly decreased under nitrogen limitation and increased under phosphorus and light limitation, and at high *p*CO_2_. The second functional cluster was defined by chlorophyll‐a and phycocyanin per cell. Both traits responded similarly to each condition, except under phosphorus limitation where chlorophyll‐a per cell decreased, while phycocyanin increased. Nitrogen limitation caused a strong decrease in these two traits while they increased under light limitation. The overall responses to high *p*CO_2_ were comparable to the control conditions. The third cluster was formed by cell size (FSC). Nitrogen limitation increased cell size, while light and phosphorus limitation reduced cell size. Lastly, the fourth functional cluster was represented by the ratio of phycocyanin over chlorophyll‐a. Nitrogen limitation reduced the phycocyanin:chlorophyll‐a ratio compared to the control, while light, phosphorus limitation and high *p*CO_2_ tended to increase this ratio. Phycocyanin fluorescence was higher than chlorophyll‐a fluorescence under light and phosphorus limitation. Lastly, we found a negative correlation between cell size and gas vesicle content (*r* = −0.48; *p* < 0.001; Figure S3).

### 3.2 Individual‐Level Based Functional Space

**FIGURE 3:**
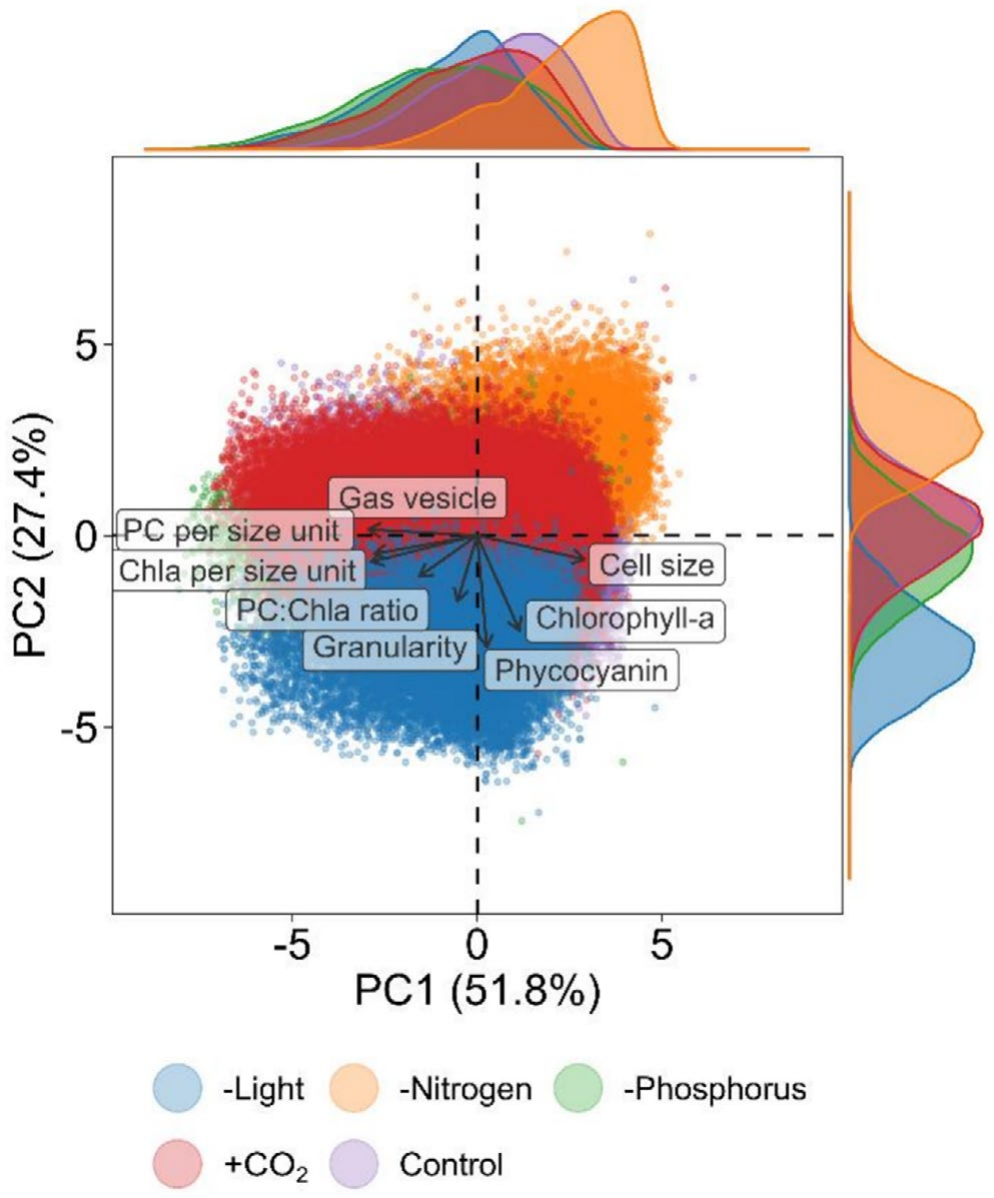
Principal Component Analysis on the eight individual‐level functional traits of *Microcystis*. The PCA represents the functional space of the tested *Microcystis* strain, where each colour represents a set of specific treatment conditions including reference conditions as the control (purple), high *p*CO_2_ (+CO_2_; red), and limitation by nitrogen (orange), phosphorus (green) and light (blue). The density curves in the margin represent the distribution of the functional space of *Microcystis* for each condition along the first two dimensions of the PCA. Each point in the scatter plot represents an individual *Microcystis* cell. The length of the arrows indicates the correlation of the functional traits with the principal components.

The dimension reduction of the eight functional traits by the PCA produced four eigenvalues capturing 100% of the functional variability. The first two PCA axes represented 79.2% of the total variance (Figure 3; 51.8% and 27.4% for axis 1 and axis 2, respectively). Overall, the morphological and morphophysiological traits (i.e., cell size, gas vesicle content, phycocyanin per size and chlorophyll‐a per size) were closer associated with axis 1 while physiological traits (i.e., granularity, phycocyanin and chlorophyll‐a content) were better associated with axis 2 (Table S1). The PCA captured a negative association between cell size and gas vesicle content, which suggests a trade‐off between these two traits. The functional differentiation across treatments was illustrated by the density curves on the margin. The difference in functional response was more apparent along the physiological axis than the morphological axis. This suggests a more diverse physiological response of *Microcystis* under environmental gradients than morphological responses, particularly between nitrogen and light limitation, thereby underscoring the importance of chlorophyll‐a and phycocyanin in capturing the physiological differences within the integrated phenotype. Other axes combinations capture large trait variation which may impact the functional space despite not reaching statistical significance (Figure S5).

### 3.3 Functional Space Across Treatments

#### 3.3.1 Characterisation of the Functional Spaces

**FIGURE 4:**
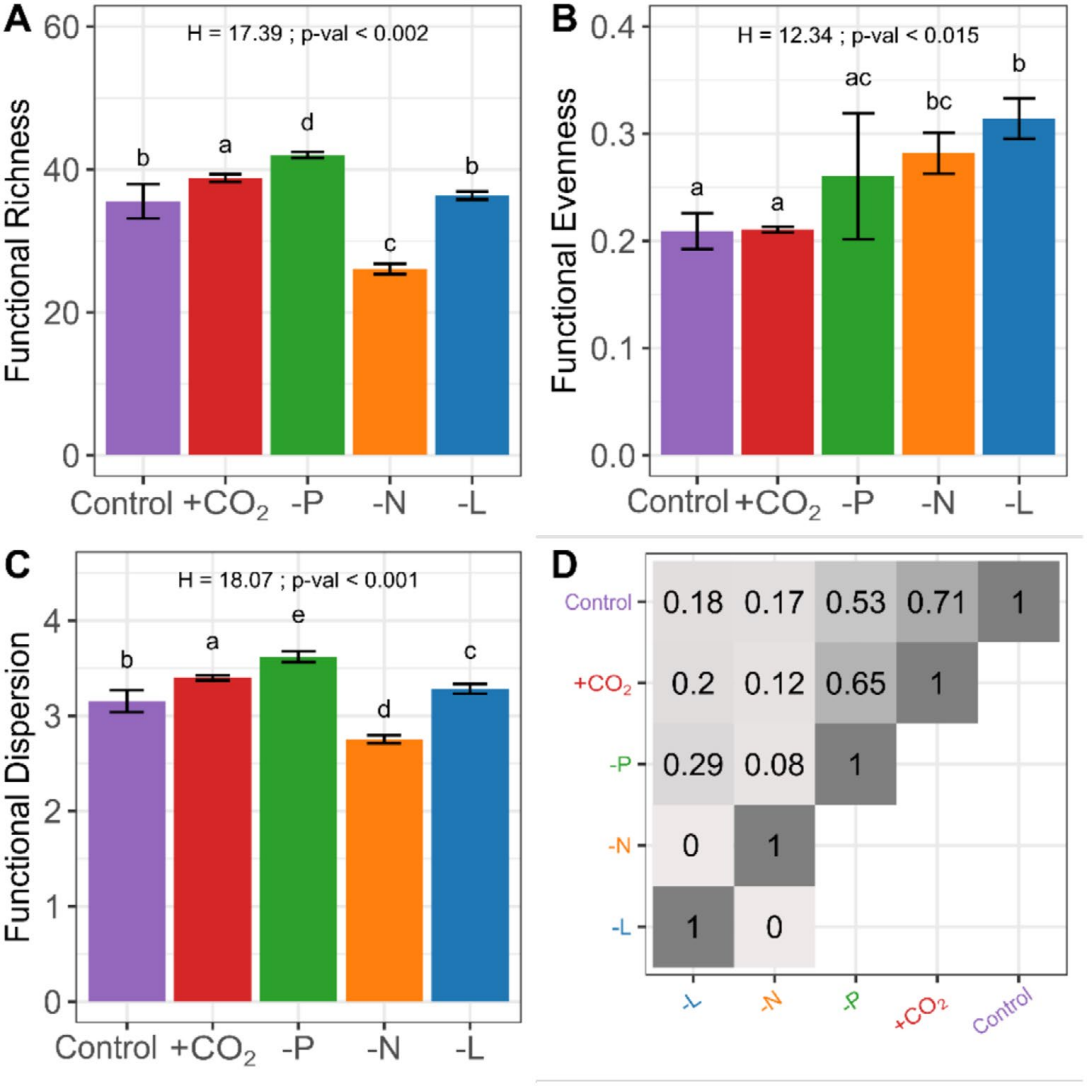
Individual‐level functional diversity indices of a *Microcystis* strain under different treatments, with (A) functional richness, (B) functional evenness and (C) functional dispersion. The treatments include the control (Cont), high *p*CO_2_ (+CO_2_), phosphorus limitation (‐P), nitrogen limitation (‐N) and light limitation (−L). The letters indicate significant differences between groups based on pairwise comparison with the Kruskal–Wallis and Conover tests. (D) Jaccard similarity index between pairs of functional space. Light grey shade (*J* = 0) corresponds to absence of similarity (i.e., no overlap between the functional space of two treatments) while dark grey shade (*J* = 1) corresponds to exact similarity (i.e., no difference between the functional space of two treatments).

Principal component analysis revealed strong phenotypic plasticity in *Microcystis* across treatments (Figure 3). The variation in the integrated functional responses significantly affected functional diversity, as reflected in richness, evenness and dispersion (Figure 4). Functional richness differed across treatments (H = 17.4; *p* < 0.002) and was lowest under nitrogen limitation and highest under phosphorus limitation. Compared to control, functional richness increased under phosphorus limitation and high *p*CO_2_ but declined under nitrogen limitation. Light limitation showed no significant differences. Functional evenness ranged from 0.21 (control) to 0.34 (light limitation), displaying significant differences (H = 12.3; *p* < 0.015). Light limitation produced a more homogeneous distribution of the individuals in the trait space compared to the control on *p*CO_2_, while nitrogen and phosphorus limitation yielded intermediate values. Functional dispersion varied significantly across treatments (H = 18.1; *p* < 0.001) and ranged from 2.75 (nitrogen limitation) to 3.62 (phosphorus limitation). Dispersion was the highest under phosphorus limitation, high *p*CO_2_, and light limitation, and lowest under nitrogen limitation.

#### 3.3.2 Functional Similarity Under Environmental Changes

Similarities between the functional spaces ranged from high (control vs. high *p*CO_2_) to none (nitrogen limitation vs. light limitation) (Figure 4d). The large range in the Jaccard index indicated high plasticity and multi‐trait variation of *Microcystis* in response to the treatments. Because of their high Jaccard index (*J* = 0.71), the high *p*CO_2_ treatment and control conditions are likely to have a comparable functional response. The control conditions, high *p*CO_2_ and the phosphorus limitation treatment also shared more than half of their functional space (*J* = 0.53 and *J* = 0.65, respectively). In contrast, the functional space of *Microcystis* under light and nitrogen limitation was dissimilar to any other condition (*J* < 0.30), which indicates that *Microcystis* produces distinct functional responses under these conditions.

### 3.4 Functional Assessment of Natural Communities

**FIGURE 5:**
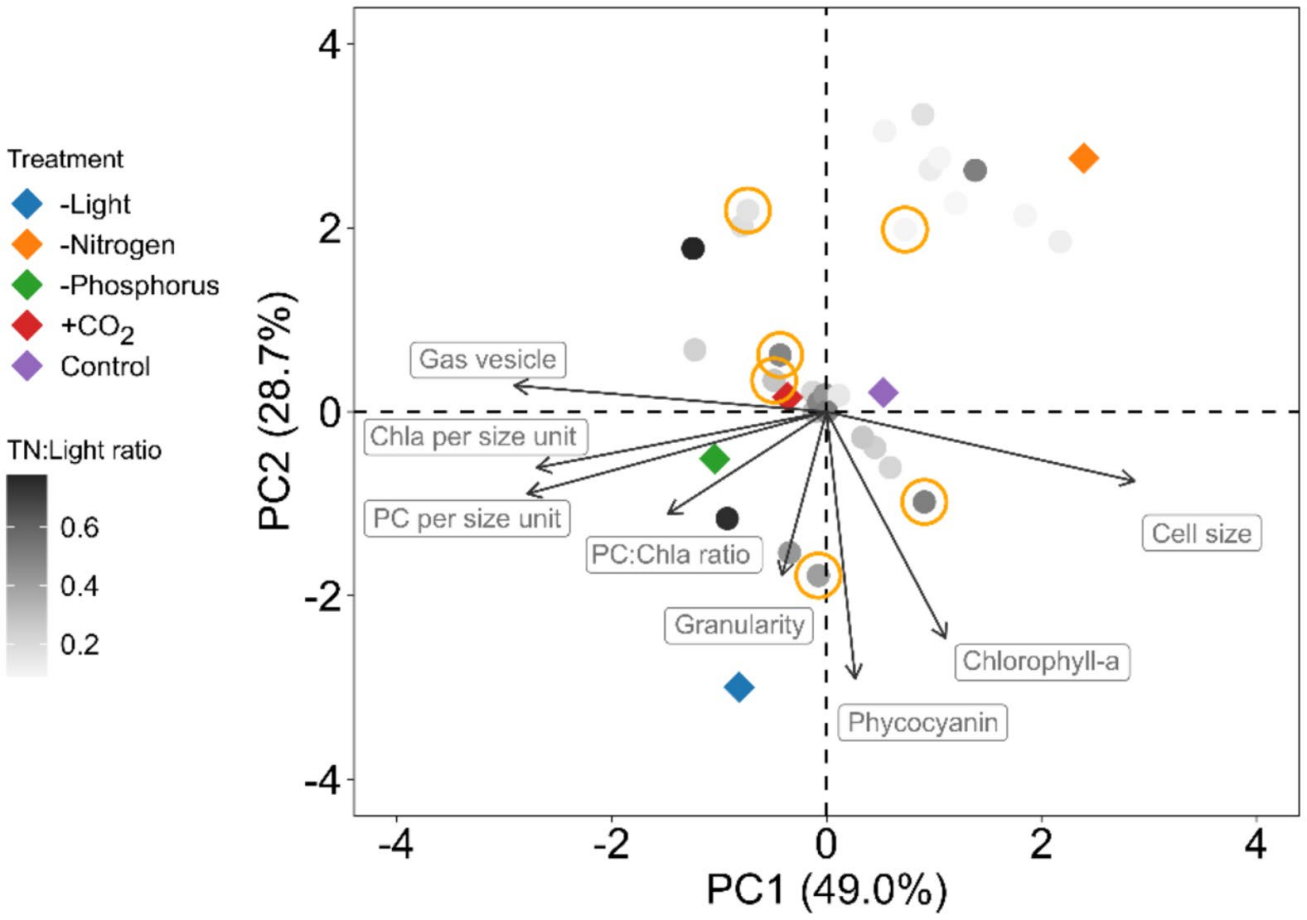
Functional assessment of cyanobacterial natural communities of lake Grote Plas and the tested *Microcystis* strain under different treatments. The PCA describes the average integrated phenotype, based on the individual particles‐level for the *Microcystis* strain, and the corresponding cyanobacterial cell‐size fraction (i.e., 0.5–10 μm) for natural communities in the Grote Plas (Figure S9). The average integrated phenotypes to treatment conditions in the *Microcystis* cultures are indicated by the large coloured circles, with control (purple), high *p*CO_2_ (+CO_2_; red) and limitation by nitrogen (orange), light (blue) and phosphorus (green). The cyanobacteria from natural communities in the Grote Plas are indicated by the smaller circles in scaling from light grey (low total nitrogen: Light ratio) to black (high total nitrogen: Light ratio). The symbols circled in orange represent dates with confirmed nitrogen limitation identified in bioassays.

Integration of traits in natural samples from the individual‐level to the community‐level revealed that the phenotypes captured by the culture of *Microcystis* are representative of the phenotypes of the cyanobacterial populations in lake Grote Plas (Figure 5). Specifically, the grey scaled symbols representing the field data fall in the same range as the coloured symbols of the culture experiments. The alignment of the natural communities with the fingerprints based on a single *Microcystis* strain indicated that trait variation in the lake was mostly associated with light and, particularly, nitrogen that showed most distinct responses. Higher phycocyanin and chlorophyll‐a content and cellular granularity were associated with the light‐limited phenotype, while larger cell size and higher phycocyanin:chlorophyll‐a ratio were associated with nitrogen limitation. The integrated phenotype of the natural communities matched with the envelope of integrated phenotypes defined by the laboratory experiment. Notably, the phenotypes of the natural communities observed under low nitrogen:light ratio matched with those obtained under nitrogen limitation in the experiment. Bioassays confirmed nitrogen limitation at six sampling dates (Figure 5; Figure S6), which corresponded to lower and intermediate nitrogen:light ratios. No phosphorus limitation was confirmed by the bioassays nor the TN:TP ratios from the field data (Figures S6 and S7).

## 4 Discussion

Our results showed that resource availability shapes the functional response of *Microcystis* at both the single‐trait and multiple‐traits levels, with the latter producing a broad range of integrated phenotypes, with the strongest responses under nitrogen and light limitation. The plasticity of the integrated phenotype determines the potential of *Microcystis* to respond to environmental changes. Moreover, our findings show that individual level trait analyses on a single strain experiment can serve, at least partly, as a fingerprint for functional assessment of natural cyanobacterial communities.

### 4.1 Individual‐Level Trait Responses as Physiological Fingerprint

Flow cytometry provided insight into intraspecific trait variation in phytoplankton by enabling measurements of eight functional traits across over 200,000 cells including cell size, pigment content, granularity (a proxy of cellular complexity and gas vesicle) and gas vesicle volume (granularity normalised by cell volume). Each functional trait presented a large array of responses confirming high trait variation within genotypes (Forsman 2015). Distinct trait responses to environmental conditions partly explain this variation (Figure 2; Figure S8). Our findings on pigment content, cell size and gas vesicle content were largely comparable to mean trait response based on traditional bulk biomass measurements from Duan et al. (2021), which confirms the potential for using individual level trait responses based on flow cytometry to infer integrated phenotypes. We only observed a difference for cell size responses to phosphorus limitation, where we report a decrease while earlier findings showed an increase. This likely resulted from using different cell size proxies, which was light scattering in our study and biovolume per cell in earlier work (Duan et al. 2021).

Cellular pigments contents were the most plastic traits, with higher contents under light limitation (Figure 2). These findings align with earlier findings on bulk responses. For example, under low light conditions, *Microcystis* upregulates pigment content to maintain photosynthesis (Bañares‐España et al. 2013). In contrast, nitrogen limitation reduces pigment synthesis, particularly phycocyanin, lowering the phycocyanin:chlorophyll‐a ratio (Allen 1984; de Van Waal et al. 2010). Nitrogen limitation affects accessory pigments, like phycocyanin or other compounds, more than major photosynthetic pigments (e.g., Litchman et al. 2002). Furthermore, nitrogen starvation induces oxidative stress causing pigment degradation for nitrogen utilisation, which decreases phycocyanin and chlorophyll‐a content (Schwarz and Forchhammer 2005). Similar to our findings, earlier studies have also shown that cellular contents of chlorophyll‐a affected by phosphorus limitation (e.g., Guedes et al. 2019).

Contrariwise to pigment contents and cell size, granularity (proxy for gas vesicles and organelles) and gas vesicle content normalised by cell volume did not show strong intraspecific variations within treatments (Figure 2). Overall, we observed decreased gas vesicle contents under nitrogen limitation (Duan et al. 2021), which was expected as gas vesicles are nitrogen‐rich (Walsby 1994). Limited functional trait variation suggests that individuals converge towards similar strategies under given conditions. Our findings show that pigment contents and cell size vary between cells within treatments, indicating weak synchronisation of strategies. In contrast, the minimal variation in granularity and associated traits across cells within treatments suggests a consistent physiological response among cells, which may reflect energetic constraints rather than synchronisation.

Overall, buoyancy is determined by several traits, and increases with gas vesicle volume (Brookes 2000), decreases with carbohydrate content that serves as ballast (Thomas and Walsby 1985; Visser et al. 1995), and is furthermore influenced by cell or colony size (Xiao et al. 2012), suggesting a potential trade‐off. This is confirmed by our PCA analysis on 200,000 cells from a natural *Microcystis* population which showed a negative correlation between cell size and gas vesicles (Figure 3). This potential trade‐off was mainly driven by the decrease in gas vesicle volume under nitrogen limitation, linked to larger cells caused by arrested growth and carbon accumulation (Duan et al. 2021). Thus, nitrogen limitation likely enhances sinking rates as a physiological consequence of reduced gas vesicle and increased carbon content. However, since we obtained this trade‐off from a single *Microcystis* genotype, it remains unclear whether the relationship occurs across genotypes or species. Hence, additional genotypes need to be assessed to determine the consistency of this trade‐off within *Microcystis* populations.

### 4.2 Integrated Phenotype and Functional Diversity

Integration of multiple individual‐level trait responses revealed the integrated phenotype of a single *Microcystis* strain (Figure 3). The integrated phenotype reveals responses beyond those observed in single traits, with chlorophyll‐a and phycocyanin contributing strongly to the physiological differences within the multidimensional trait space. For example, although the individual trait responses towards high *p*CO_2_ levels were like the control conditions (Figure 2), it led to a different integrated phenotype (Figure 4). Functional diversity was high under high *p*CO_2_ and phosphorus limitation as these conditions led to a large overall range of distinct phenotypes (i.e., high functional richness and dispersion, respectively). This indicates that environmental heterogeneity under these conditions drives greater phenotypic heterogeneity, allowing *Microcystis* cells to adopt a diverse set of strategies involving variation in cell size, gas vesicle and pigment contents (Figure 3). These findings are in line with previous studies stating that resource limitation induces phenotypic plasticity (Fontana et al. 2018; Schreiber et al. 2016). In contrast, functional diversity is strongly reduced (i.e., low functional richness and low functional dispersion) under nitrogen limitation, resulting in a narrow range of phenotypes that mostly reflected reduced pigment contents indicating pigments breakdown (Schwarz and Forchhammer 2005). The increased pigment contents under light limitation indicate adaptation to low‐light conditions, where high trait plasticity for light acquisition grants a competitive advantage (Schwaderer et al. 2011).

The high functional dissimilarities between the control, light limitation and nitrogen limitation treatments determine how a single strain can adjust to changing environments. The contrasted responses observed from the individual level deviate from the mean field theory (Macarthur and Levins 1967), which assumes uniform responses. Therefore, mean trait values, which are often used to assess functional diversity, may not explain phytoplankton species dynamics. Furthermore, the ability of *Microcystis* to produce various functional responses under light and nitrogen limitation may provide a competitive advantage.

The heterogeneity in cellular responses within a genotype occurs in heterotrophic bacteria and may arise due to stochastic gene expression or differential cellular metabolic networks activities (Gasperotti et al. 2020). Resource limitation may increase phenotypic heterogeneity in clonal populations, potentially serving as an adaptive strategy in low‐resource and fluctuating environments (Gasperotti et al. 2020; Opalek et al. 2022). Our study shows that phenotypic heterogeneity can be high in cyanobacteria and possibly support blooms in dynamic environments.

### 4.3 Scaling From Individual to Community Level Responses

The integrated phenotypes observed in the single *Microcystis* strain are representative of the trait expressions range present in the cyanobacterial communities of lake Grote Plas (Figure 5 and Figure S7). Notably, mapping individual level responses of *Microcystis* strains and natural cyanobacterial populations and communities onto a shared trait space revealed a continuous phenotypic variation along a nitrogen: light ratio (Figure S9). This finding contrasts with traditional mean field theory of discrete phenotypes, which overlooks the plasticity and environmental responsiveness of phytoplankton populations. Pigment responses especially capture this as they are sensitive to both light and nitrogen limitation, which marked the trait space boundaries of the trait space as indicated by the experimental findings (Figures 2 and 5). These results underscore the use of functional traits from laboratory experiments to predict natural community responses (Edwards et al. 2013). This match was particularly strong for nitrogen limitation, where natural communities resembled low nitrogen: light ratios and corresponded to the integrated phenotype of nitrogen‐limited cultures. Moreover, in some cases, the phenotypes under nitrogen‐limited conditions reflected those observed under control or phosphorus‐limited conditions. Furthermore, the intermediate phenotypes produced by phosphorus limitation and high *p*CO_2_ highlight that multiple environmental factors modulate trait expression, contributing to the continuous phenotypic variation observed in the field populations (Figure S9). Differences in responses of field populations and laboratory studies could also result from genotypic variations, as *Microcystis* genotype composition often varies during a bloom (Chun et al. 2020; Kardinaal et al. 2007; Liu et al. 2016). Our findings reveal that trait responses may vary even within a single strain, underscoring the complexity of cyanobacterial functional responses from cells to communities. Biotic interactions also shape phytoplankton functional traits and their individual level integrated phenotype. Grazing by zooplankton, for example, benefits smaller cells and enhances colony formation of filaments (Xiao et al. 2018). Infections by viruses and fungal parasites add further complexity, as they may selectively lyse dominant phytoplankton genotypes and associated phenotypes (Suttle, 2007; Sime‐Ngando, 2012). Meanwhile, competition for nutrients and light structures trait values among species and genotypes, favouring those able to optimise their physiology (Kardinaal et al. 2007). Moreover, environmental conditions vary substantially, resulting in niche shifts and associated changes in dominant phenotypes (Tromas et al. 2018). Despite this wide range of factors, our findings show a consistent response of the integrated phenotype of *Microcystis* populations that reflect responses of light and, particularly, nitrogen limited laboratory cultures. This suggests a potential use of individual level responses to infer the physiological state of natural populations and communities.

The application of functional fingerprints to natural cyanobacterial populations and communities illustrates how functional responses can be integrated across ecological scales, from individuals to community levels (Chacón‐Labella et al. 2023; Fontana et al. 2021; Litchman et al. 2007). This approach helps us understand how abiotic factors shape community structure and functioning. However, in our current study, we only obtained the functional fingerprints from a single *Microcystis* strain. Moreover, our field study excluded large colonies by applying a 10 μm threshold, thereby focusing only on single cells and small colonies. Consequently, our approach did not capture colony‐level traits that can strongly influence buoyancy, nutrient uptake, and grazing resistance. The strong overlap in functional responses between the *Microcystis* strain and the < 10 μm field assemblage indicates that *Microcystis* single‐cell intraspecific variation spans the combined inter‐ and intraspecific variation in the field, indicating that the contribution of small colonies to the < 10 μm fraction is negligible. While our study captures individual‐level intraspecific trait variation, it underestimates the intraspecific trait variation that occurs across multiple genotypes which are known to be significant (e.g., Brandenburg et al. 2018; Ryderheim and Kiørboe 2024; Wilson et al. 2005). Future studies should thus combine multiple recently isolated strains, to reduce artefacts of long‐term culture maintenance and increase the ecological relevance of experimentally derived functional fingerprints (e.g., Willis et al. 2022). Furthermore, assessments including multiple strains, additional species, and a broader range of traits would improve functional fingerprints across genotypes, species and populations, to predict functional responses of phytoplankton communities.

## Author Contributions

Arnaud P. Louchart and Dedmer B. Van de Waal conceived the idea of this study. Zhipeng Duan and Dedmer B. Van de Waal designed the experiment on *Microcystis*, and Zhipeng Duan performed the experiment. Zhipeng Duan and Suzanne M.H. Naus‐Wiezer performed flow cytometry analyses. Annemieke M. Drost and Chaohong Lin sampled Lake De Grote Plas and conducted the bioassay experiments. Arnaud P. Louchart performed flow cytometry clustering and statistical analyses. Arnaud P. Louchart, Elena Litchman. and Dedmer B. Van de Waal interpreted the results. Arnaud P. Louchart and Dedmer B. Van de Waal wrote the first drafts of the manuscript. All authors reviewed all versions of the manuscript.

## Funding

Arnaud P. Louchart, Annemieke M. Drost, Chaohong Lin and Dedmer B. Van de Waal are funded by the European Union (ERC, BLOOMTOX, project number 101044452). Views and opinions expressed are however those of the author(s) only and do not necessarily reflect those of the European Union or the European Research Council Executive Agency. Neither the European Union nor the granting authority can be held responsible for them. Zhipeng Duan is funded by the National Natural Science Foundation of China (NSFC, project number 32301373).

## Conflicts of Interest

The authors declare no conflicts of interest.

## Supporting information


**Data S1:** ele70348‐sup‐0001‐DataS1.docx.

## Data Availability

All data supporting and code the findings of this study are available at DataverseNL (doi: 10.34894/9X9YMO). PhytoCytoTraits code is available on Zenodo (doi: 10.5281/zenodo.14925458).
