# Supplementary material for: Individual‐Level Trait Responses in Cyanobacterial Populations and Communities
Source: Ecol Lett. 2026 Feb 23;29(2):e70348. doi: 10.1111/ele.70348 (PMC12929705; doi:10.1111/ele.70348)
Supplement: Supplementary file 1 — Data S1: ele70348‐sup‐0001‐DataS1.docx. [file ELE-29-0-s001.docx]

**Supplementary information: Individual-level trait responses in cyanobacterial populations and communities**

Arnaud P. Louchart, Annemieke M. Drost, Chaohong Lin­, Suzanne M.H. Naus-Wiezer, Zhipeng Duan, Elena Litchman, Dedmer B. Van de Waal

**Content**

[**Appendix 1: Description of field samples** 2](#_Toc217147097)

[**Appendix 2: Description of bioassays** 2](#_Toc217147098)

[**Supplementary figures S1-S5** 3](#_Toc217147099)

[**Supplementary table** 11](#_Toc217147100)

# **Appendix 1: Description of field samples**

We collected 30 L surface water samples approximately 5 meters from the lake edge. After collection, we gently homogenized the water in a bucket and subsampled it for total nitrogen (TN), total phosphorus (TP), and dissolved inorganic nutrients. We also conducted *in situ* measurements. We measured light intensity just below the surface and at 20 cm depth using a LI-250A Light Meter (LI-COR, Lincoln, NE, USA). To determine TN and TP, 12 mL of each sample was acidified with 12 μL of concentrated hydrochloric acid (HCl, 37%, analytical grade) and stored at 4°C in the dark until analysis. Dissolved inorganic nutrient samples were immediately filtered in the field using a 0.45 µm 25 mm cellulose acetate filter (Avantor, PA, U.S.A.) and samples were stored at −20°C. Analyses of dissolved inorganic nutrients, including ammonium (NH₄⁺), nitrate (NO₃⁻), nitrite (NO₂⁻), and phosphate (PO₄³⁻), as well as TN and TP, were conducted using a QuAAtro39 Auto-Analyzer (SEAL Analytical Ltd., Southampton, UK).

# **Appendix 2: Description of bioassays**

Bioassay experiments were conducted to identify nitrogen and phosphorus limitation from the field samples. Bioassays began the morning of the day following field sampling. Lake water was homogenized and filtered through a 200 μm mesh to avoid grazing by large zooplankton. One-liter aliquots were incubated in glass jars placed in temperature-controlled water baths for four days (Thursday – Monday). Light intensity was maintained at 100 ± 5 μmol photons m⁻² s⁻¹. Photoperiods were set at 17 h for the first four bioassays (June – July bioassays) and 15 h for the last three bioassays (August – September bioassays), each with a 1 h gradual light ramp-up and ramp-down to reflect natural light conditions of the lake. This resulted in 15 h and 13 h of full light intensity, respectively. Incubation temperatures of the water bath were based on *in situ* measurements taken between 09:00 and 10:00 am on sampling days, resulting in respective temperatures of 20.8, 24.8, 25.2, 21.0, 19.6, 21.0 and 19.2°C for the seven bioassays. Outcomes of all bioassays are shown in Figure S6.

Four treatments were applied in quadruplicate: (1) control (no addition), (2) nitrogen addition (50 μM KNO₃), (3) phosphorus addition (3 μM KH₂PO₄), and (4) combined nitrogen and phosphorus addition (50 μM KNO₃ and 3 μM KH₂PO₄). Nutrient additions were selected to support at least a fourfold increase in phytoplankton biomass, based on assumptions that 1 μg L⁻¹ chlorophyll-a requires approximately 1 μmol L^-1^ particulate organic nitrogen. In July, chlorophyll-a concentrations were 10 μg L⁻¹, and 50 µM of KNO_3_ was added to support phytoplankton growth. Accordingly, KH_2_PO_4_ was added at a concentration of 3 µM to approximate the Redfield ratio (N:P = 16:1). Phytoplankton biomass on days 0 and 4 was estimated by chlorophyll-a fluorescence using the PHYTO-PAM (Heinz Walz GmbH).

# **Supplementary figures S1-S5**


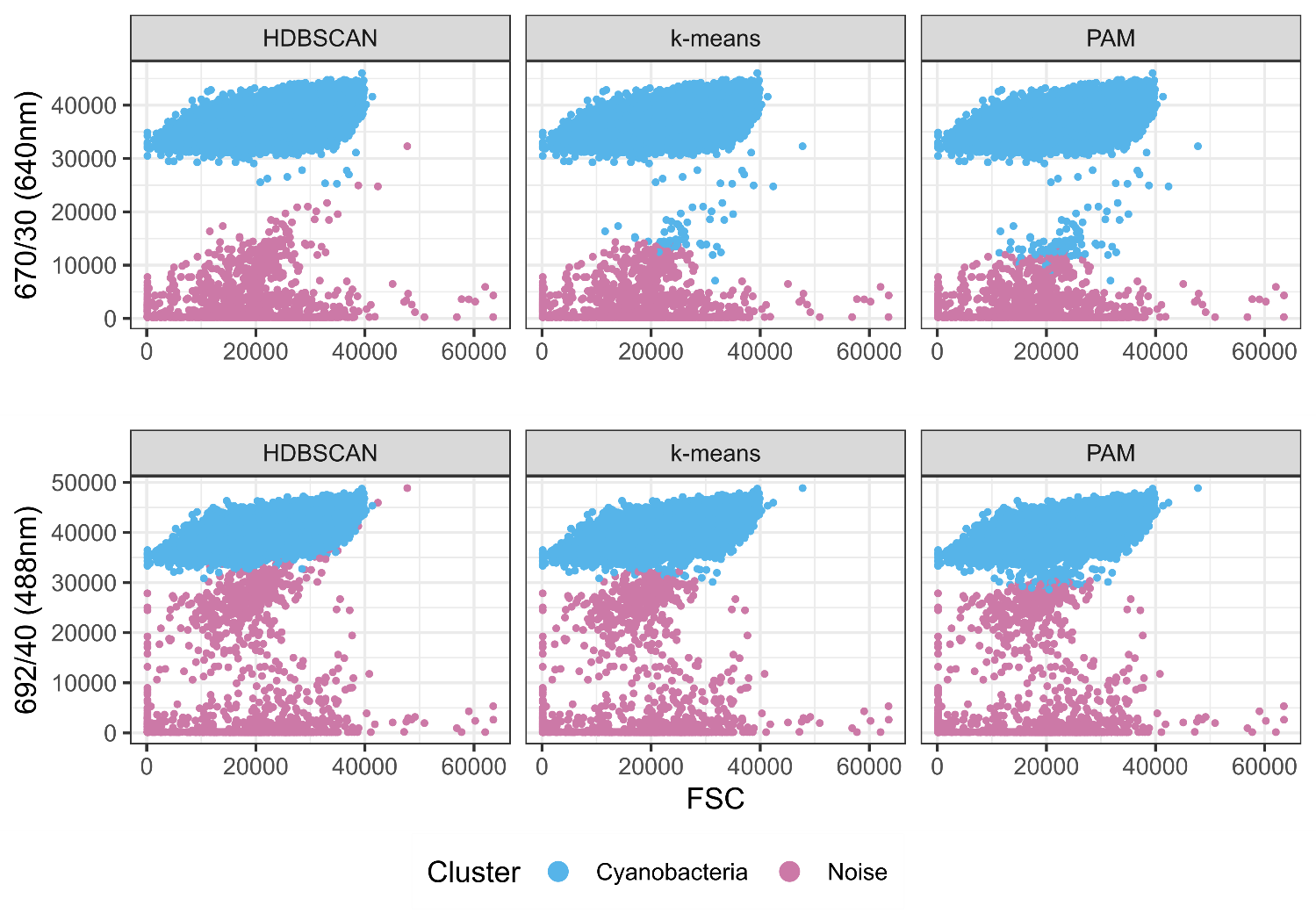
Figure S1: Example of results from the three different unsupervised clustering methods (HDBSCAN, k-means, PAM) available in the application PhytoCytoTraits. The sample analysed corresponds to replicate 1 of the high *p*CO_2_ treatment. Axes represent optical traits from the flow cytometer. In terms of ecological relevance, Forward Scatter (FSC) estimates cell size, 670/30 (640 nm) provides phycocyanin fluorescence, and 692/40 (488nm) provides chlorophyll-a fluorescence. HDBSCAN performed better recognition of cyanobacterial cells compared to k-means and PAM methods. In the HDBSCAN method, clusters required at least 1% of the total particles per datafile. The radius (*ε*) was automatically calculated using the *k*-nearest neighbor distance (*k* = 5).


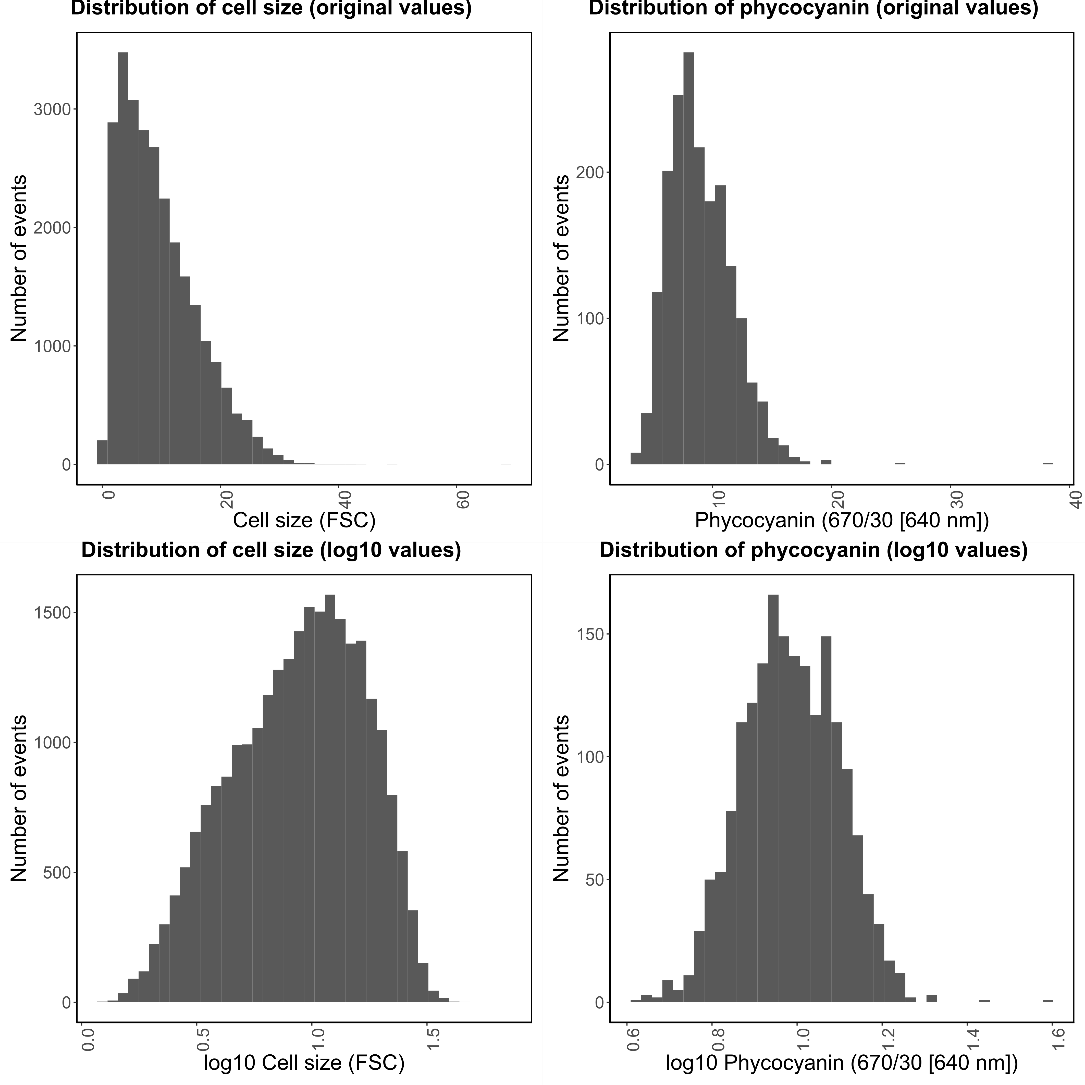


Fig. S2: Example of trait value distributions from the original data (top) and log_10_-transformed data (bottom) for cell size (left: control treatment, replicate 1) and phycocyanin (right: nitrogen-limited treatment, replicate 3).


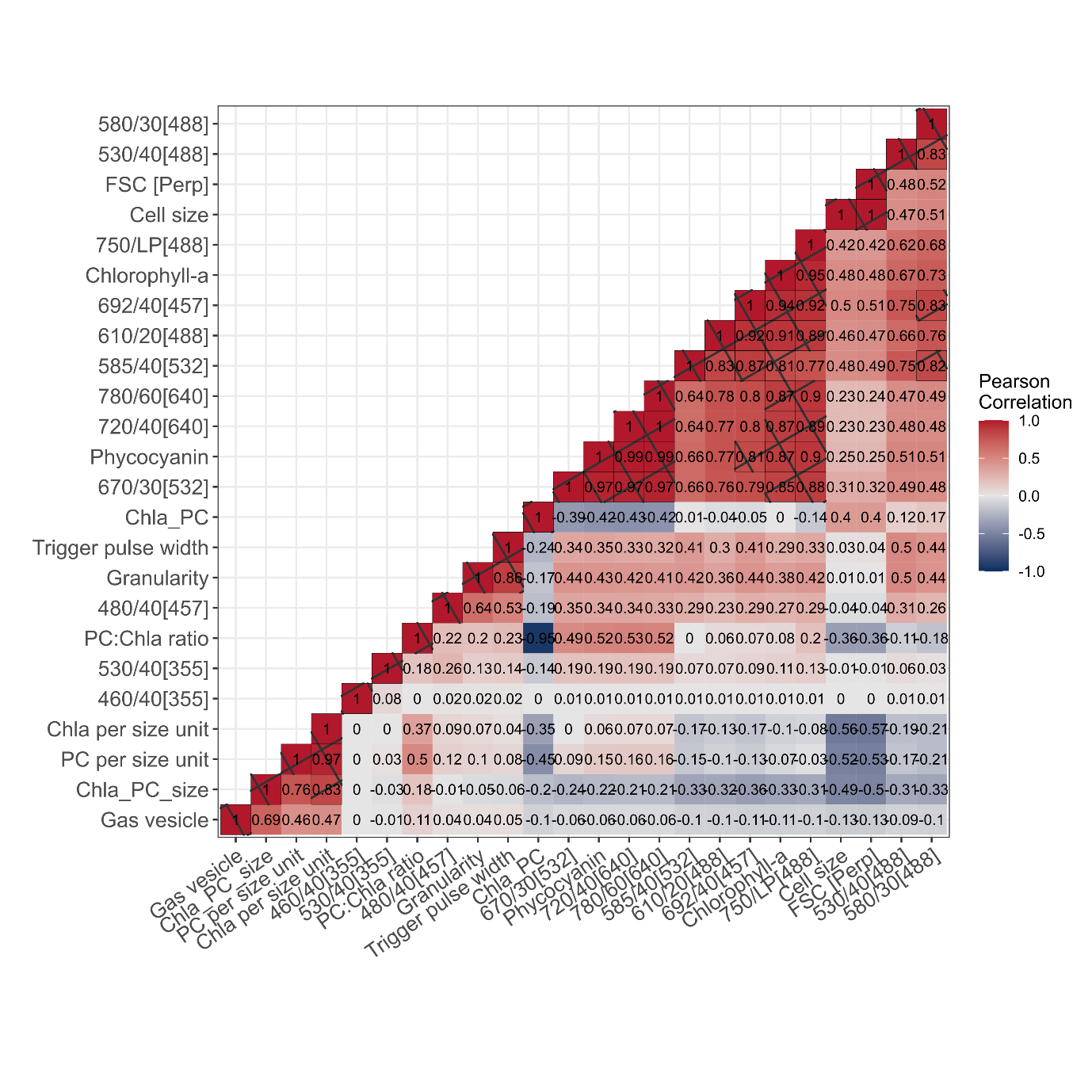
Figure S3: Spearman correlation matrix of the optical traits derived from flow cytometry scatter and fluorescence signals. Traits originate from the signal intensity obtained by the combination of filters (wavelength of emission/bandwidth) associated to a laser (wavelength indicated between brackets). The number in each tile represents the Pearson correlation coefficient for each pair of traits. The gridded tiles represent high collinearity between traits (r > 0.8).


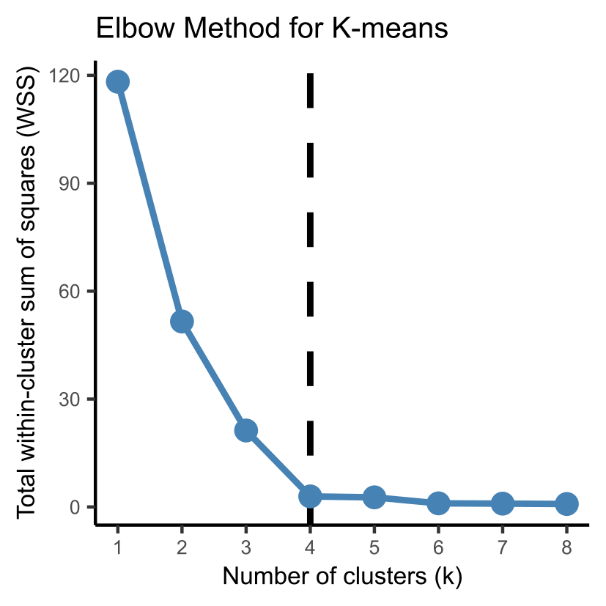


Fig. S4: Determination of the optimal number of clusters for k-means clustering based on the eight functional traits. Total within-cluster sum of squares (WSS) is plotted against the number of clusters (k). The “elbow” in the curve indicates the k value beyond which adding more clusters does not substantially reduce WSS, guiding selection of the most suitable number of clusters.


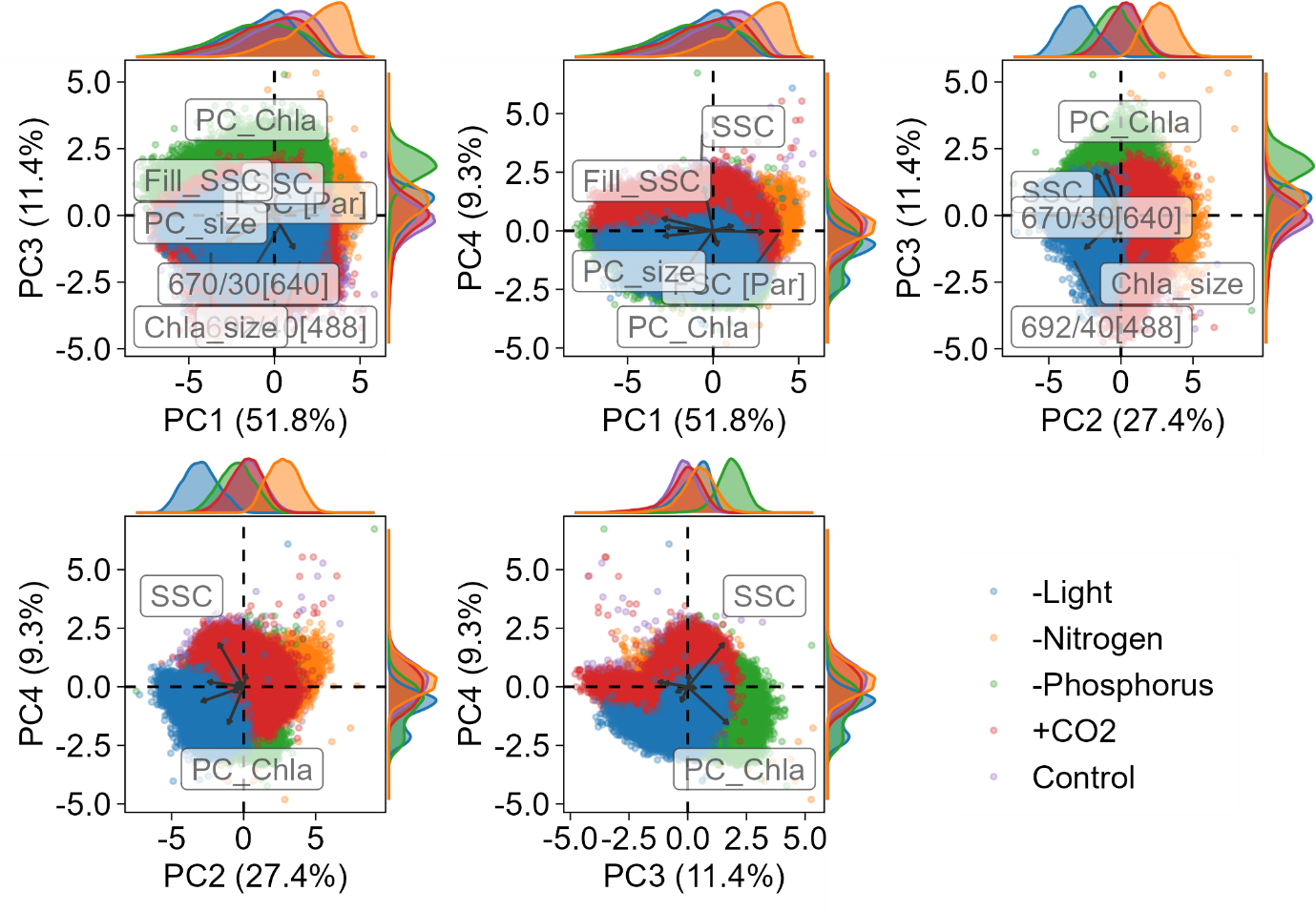
Figure S5: Principal Component Analysis on the eight individual-level functional traits based on the culture experiment with *Microcystis* sp. Each plot represents the combination of two axes and the distribution of the individuals in the factorial plane. The PCA represents the functional space of *Microcystis*, where each color represents a set of specific treatment conditions including reference conditions as the control (purple), high *p*CO_2_ (red), and limitation by nitrogen (orange), phosphorus (green) and light (blue). The density curves in the margin represent the distribution of the functional space of *Microcystis* for each condition along the first two dimensions of the PCA. Each point in the scatter plot represents a cell of *Microcystis*. The length of the arrows indicates the correlation of the functional traits with the principal components.


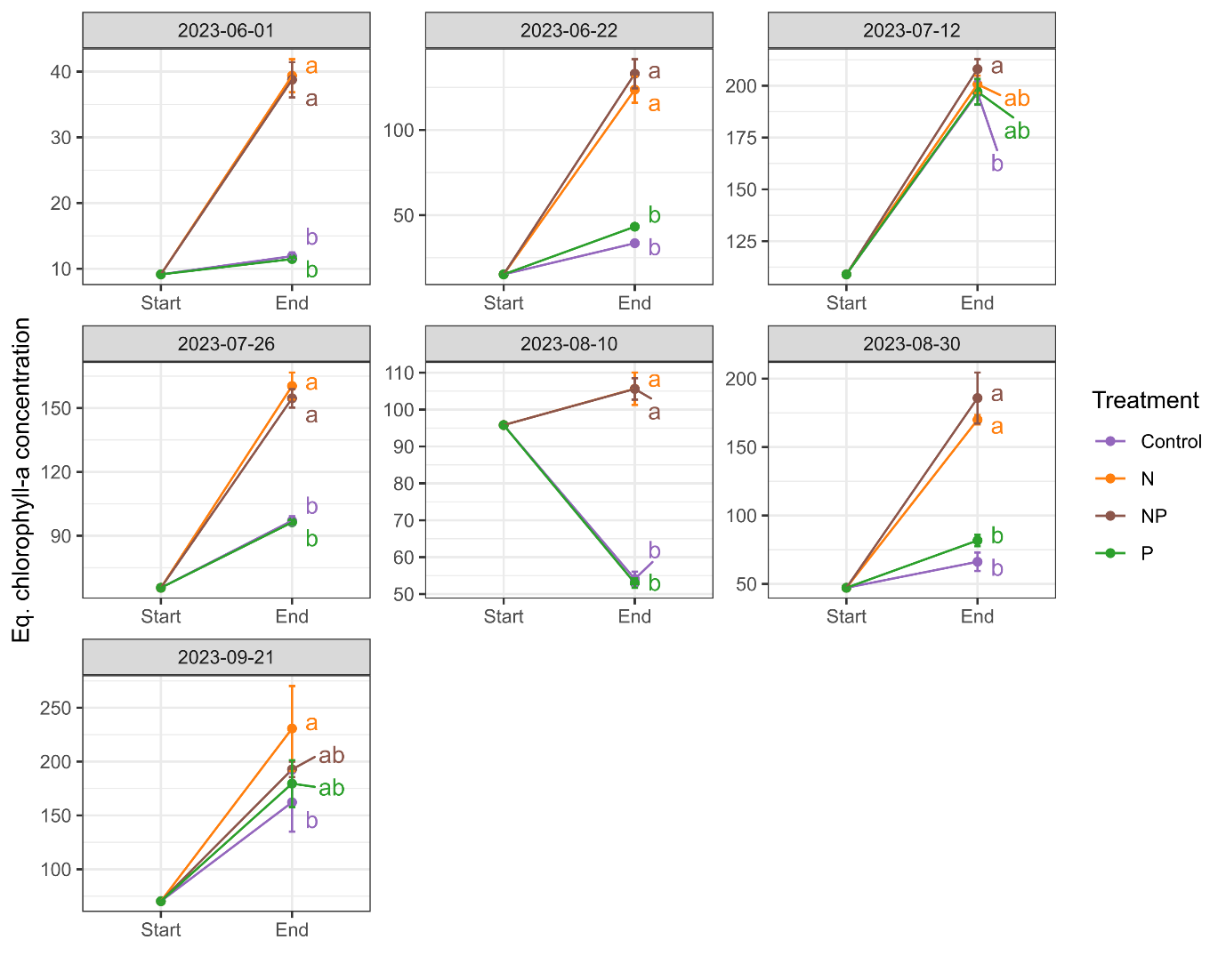


Figure S6: Nutrient bioassay results showing equivalent of chlorophyll-a concentration, as derived from the chlorophyll-a fluorescence obtained from the PhytoPAM, across seven sampling dates in 2023. Treatments with nitrogen (N) and combined nitrogen-phosphorus (NP) consistently induced highest phytoplankton growth, while control and phosphorus-only (P) treatments exhibited significantly lower responses. ANOVA and Tukey-HSD showed significant differences (p < 0.05) among treatments at the end of the experiment, which are indicated by different letters.


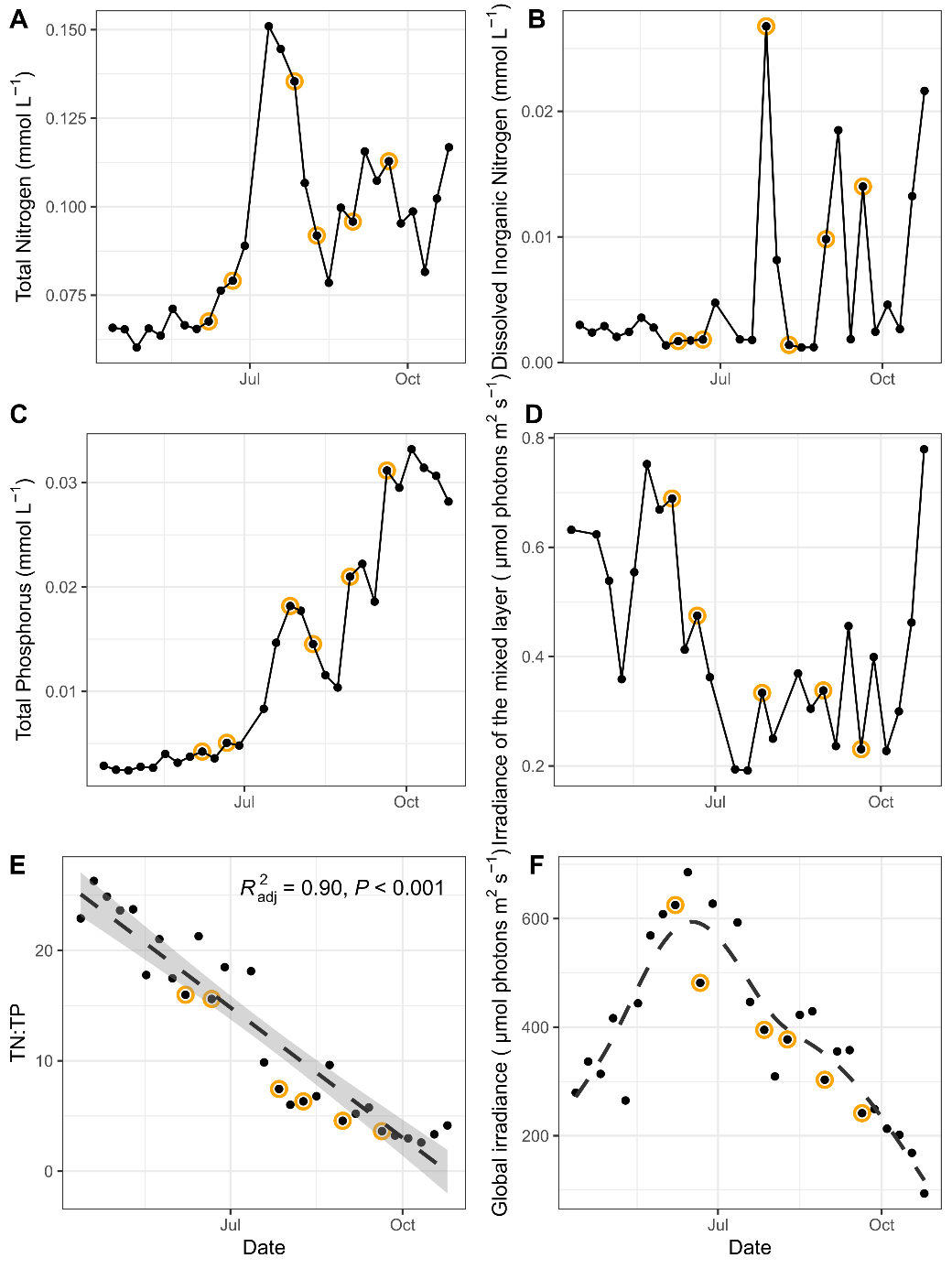


Figure S7: Dynamics of environmental parameters in lake Grote Plas in 2023 with total nitrogen (A), dissolved inorganic nitrogen (B), total phosphorus (C), Iirradiance of the mixed layer (D), TN:TP ratio (E), and the global irradiance (PAR range; µmol photons m^2^ s^-1^) recorded at the Rotterdam Airport meteorological station (Royal Netherlands Meteorological Institute; KNMI), the closest station to lake Grote Plas (F). Black dots circled in orange are dates on which nitrogen limitation was confirmed based on the bioassays.


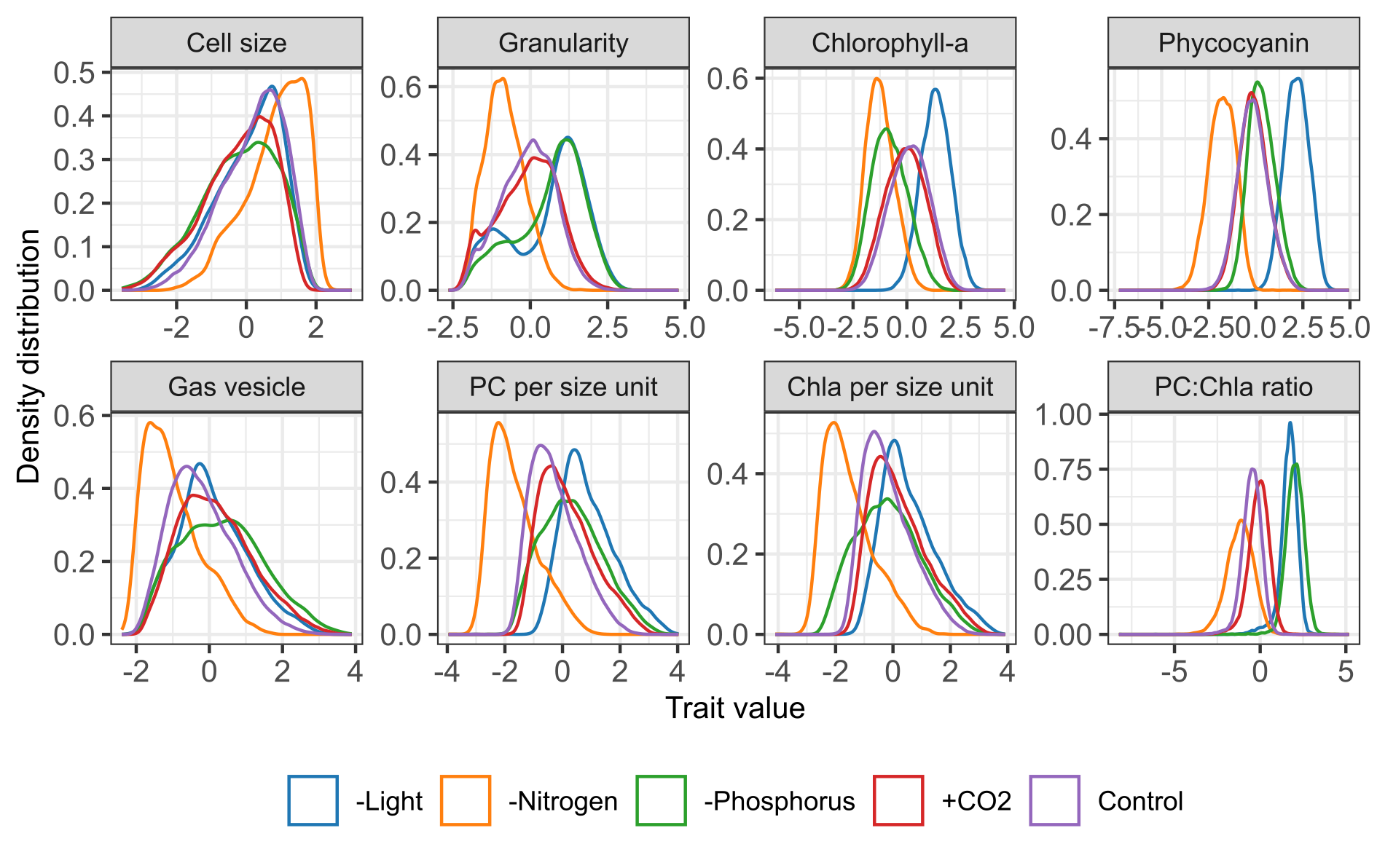


Fig. S8: Density distribution of the eight functional traits across the five treatments. The correspond to treatment conditions, with control (purple), high *p*CO_2_ (red), and limitation by nitrogen (orange), light (blue) and phosphorus (green). Each functional trait was log_10_ transformed and z-standardized.


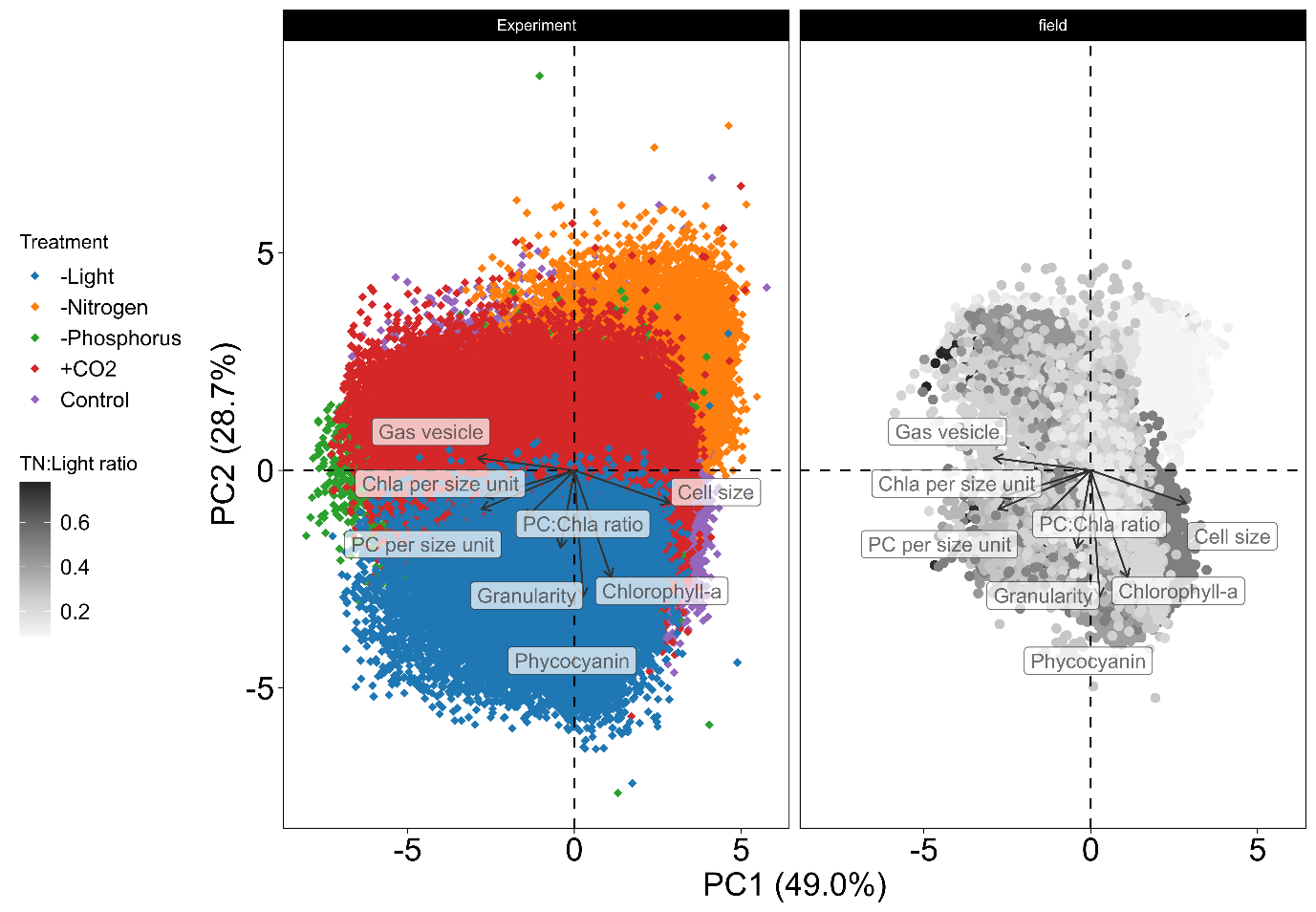


Fig. S9: Functional assessment of cyanobacterial natural communities of lake Grote Plas and *Microcystis* spp. culture under different treatments. The PCA describes all individual phenotypes from the *Microcystis* spp. culture (left panel) and from cyanobacterial size fraction of the natural communities of lake Grote Plas samples (right panel). The colors on the right panel correspond to treatment conditions, with control (purple), high *p*CO_2_ (red), and limitation by nitrogen (orange), light (blue) and phosphorus (green). The cyanobacteria from natural communities in the Grote Plas are indicated by the smaller circles in scaling from light grey (low nitrogen:light ratio) to black (high nitrogen:light ratio).

# **Supplementary table**

Table S1: Contribution of *Microcystis* spp. functional traits to PCA axes 1 and 2 and their overall contribution (sum of the contributions of axes 1 and 2). Traits highlighted in orange are morphological and morphophysiological traits. Traits highlighted in green are physiological traits.

| Trait | Comp.1 | Comp.2 | Overall explained |
| --- | --- | --- | --- |
| Size | 92.31 | 5.90 | 98.21 |
| PC_size | 92.27 | 3.65 | 95.92 |
| Phycocyanin | 0.32 | 94.18 | 94.50 |
| Gas vesicle volume | 94.41 | 0.02 | 94.43 |
| Chla_size | 86.19 | 1.56 | 87.75 |
| Chlorophyll-a | 12.74 | 70.27 | 83.01 |
| Chla_PC | 28.67 | 10.98 | 39.65 |
| Granularity | 4.87 | 32.42 | 37.29 |
